# The involvement of individuals with speech and language impairments in research: insights from a co-creation process

**DOI:** 10.1186/s40900-025-00762-8

**Published:** 2025-08-04

**Authors:** Katharina Giordano, Manja Wonschik, Juliane Leinweber

**Affiliations:** HAWK University of Applied Sciences and Arts Hildesheim, Göttingen, Holzminden Germany

**Keywords:** Co-Creation, Involvement, Speech and Language Therapy, Stroke

## Abstract

**Background:**

Involvement of end users in development processes is increasing, but there are still relatively few examples of the involvement of people with speech and language impairment (PWSLI). One reason for this is that these impairments are sometimes seen as a criterion for exclusion. The aim of this article is to identify both opportunities and challenges in a co-created technology development process with PWSLI. The project HiSSS (Hybrid and interactive speech and language therapy after stroke) has the objective of developing a hybrid teletherapy application for the treatment of speech and language impairments following a stroke. It includes a co-creation development process, engaging people with speech and language impairments and speech and language therapists. A comprehensive evaluation of the co-creation process in the HiSSS project will highlight opportunities and challenges in a co-created technology development process with PWSLI.All affiliations are captured correctly

**Methods:**

The data sources (workshop transcripts, process adaptations, team meeting protocols, researchers' reflective notes) from the co-creation process were analysed using Braun and Clarke's reflexive thematic analysis (2021). Both challenges and opportunities were identified deductively through the analysis. In a further step, the authors inductively generated themes to which they assigned the opportunities and challenges.

**Results:**

Six workshops with a total of 11 speech and language therapists and four workshops with a total of 7 people with speech and/or language impairments took place. Through thematic analysis four themes were generated: (1) Communicative limitations, (2) Researcher skills, (3) Interprofessional collaboration, (4) Organisation of participation.

**Conclusions:**

The generated themes represent dimensions of co-creation that should be considered in future technology developments. This study demonstrates the complexity of technology co-creation with PWSLI and confirms that they can be effectively involved in research and contribute meaningfully to technology development. Whether and to what extent users should be involved in research projects must be clarified as early as possible and on a case-by-case basis according to the specific objectives of the respective project. In order to take these four dimensions into account in future co-creation projects, the funding conditions must provide the necessary framework.

**Trial registration:**

The study is registered in the German Register of Clinical Trials (DRKS00030430).

**Supplementary Information:**

The online version contains supplementary material available at 10.1186/s40900-025-00762-8.

## Abstract (plain language summary)

There are still relatively few examples of the involvement of people with speech and language impairments (PWSLI) in the development process of new technologies. Some other research groups described the lack of involvement of people with communication impairments. This article shows why and how people with speech and language impairments can be involved in development processes. The HiSSS (Hybrid and interactive speech and language therapy after stroke) project aims to develop a hybrid teletherapy app for treating speech and language impairments after a stroke. The project involves people with speech and/or language impairments as well as speech and language therapists to create the new platform. Looking at how this was done will help us understand how to make new technology work well in speech and language therapy.

The data sources (workshop transcripts, process adaptations, team meeting protocols, researchers' reflective notes) from the co-creation process were analysed using Braun and Clarke's reflexive thematic analysis (2021). Both challenges and opportunities were identified deductively through the analysis. In a further step, the authors inductively generated themes to which they assigned the opportunities and challenges. Six workshops with a total of 11 speech and language therapists and four workshops with a total of 7 people with speech and/or language impairments took place. Through thematic analysis four themes were generated: (1) Communicative limitations, (2) Researcher skills, (3) Interprofessional collaboration, (4) Organisation of participation. People with speech and/or language impairment can be successfully integrated into development processes, but a methodologically designed and target group-oriented approach is necessary. It is important to decide early on how users will be involved in a research project and on a case-by-case basis. The implementation of co-creation based on these dimensions depends on the funding conditions for research.

**Supplementary Information:**

The online version contains supplementary material available at 10.1186/s40900-025-00762-8.

## Background

A stroke may be accompanied by a number of additional conditions, including impairment of speech and language. Approximately 20% of individuals who experience a stroke present with aphasia, dysarthria, and/or facial paresis [7, 53]. These impairments are associated with varying degrees of impairment in speech production and comprehension, as well as reading and writing.

As in other areas of healthcare, the potential for using technology in speech and language therapy has expanded considerably in recent years. For instance, technology is being researched and employed in the form of apps [49], virtual reality [17], and automated speech recognition (Deka et al., 2022). The benefits of technology in speech and language therapy include enhanced access to treatment, objective and personalised feedback, and increased motivation for users [9, 39]. Furthermore, research indicates that individuals who have suffered a stroke are interested in the use of technology for rehabilitation [34]. Concurrent evaluations of existing technologies demonstrate that technology acceptance is highly variable [23, 27, 37]. Even when clinicians use technology, they may feel uncomfortable implementing it what ultimately results in a limited use [13]. One potential explanation for this phenomenon may be that technologies fail to have an impact due to a lack of user involvement and consideration of user needs and preferences in the development process of new technologies [8].

Involving end users in the development of interventions and technologies has become increasingly valued in healthcare, as demonstrated by a systematic review [31] showing that of all articles on co-production and co-creation in health care from 1987 to 2020, 69.5% were published within the last four years. In addition to co-creation and co-production, terms such as co-design and participatory design are also used, sometimes even interchangeably [12]. The core idea of all these approaches is to involve stakeholders and thus view the potential end users of objects as the subjects of the development process, with equal and reciprocal relationships between professionals and end users [16, 21]. Participation can vary by type and time. This allows participants to take on different roles from listener to decision-maker [42], which can be applied throughout the research process or at specific stages. The communication between all those involved is an important component in order to enable innovation, share knowledge, and promote acceptance (Daly-Lynn et al., 2016). This becomes a challenge for groups of people with limited language-based skills due to their impairment.

Despite the growing involvement of end users in the healthcare system, there are still few examples of involving people with communication impairment. One reason for this is that communication impairments are sometimes regarded as an exclusion criterion [41]. However, in rehabilitation after stroke, communication impairments must be taken into account and involvement processes adapted accordingly. Singh et al. [41] identify potential strategies to overcome limitations in the inclusion of people with stroke, especially those with communication impairments. These strategies include involving speech and language therapists (SLTs), using generative tools to enable participation (e.g. Lego serious play, videos, mapping methods), and assessing communication profiles in order to be able to respond to them individually. These strategies can be found in co-design examples with people with communication impairments. The involvement process is typically conducted by or with the assistance of SLTs, and both spoken and written material are adapted to the linguistic abilities of the involved individuals [3, 4, 43]. Furthermore, the use of visualisation techniques to enhance comprehension and focus has been demonstrated to be beneficial [4],Pierce et al., 2024; [43]. There is a consensus that sufficient time and resources should be allocated to address the communicative needs of individuals with communication impairments, whether through additional training or support offers [3], the organisation of individual meetings [43], or the option of confirming one's own statements in the form of member checking [4]. These studies have shown that involving people with communication impairments in development processes of new technologies requires specific adaptations. Technology co-creation is a complex, usually agile process involving various disciplines [48]. Currently, there is limited knowledge regarding the factors that hinder or facilitate the overall process. To our knowledge, no analysis of an entire involvement process has yet been conducted to determine the requirements, challenges and necessary adjustments for people with speech and language impairments to participate in the development of technology.

The project HiSSS (Hybrid and Interactive Speech and Language Therapy after Stroke) has the objective of developing a hybrid teletherapy application for the treatment of speech and language impairments following a stroke. Teletherapy can be conducted synchronously and asynchronously, whereby synchronous teletherapy usually corresponds to an exchange via video conference with a therapist in an individual or group setting. Asynchronous teletherapy is the provision of information or material that enables the patient to perform exercises independently without simultaneous support from the therapist. The project's core component is a complex, web-based speech therapy program that integrates existing and innovative synchronous and asynchronous therapeutic elements. The innovative elements include the use of automatic speech recognition (ASR) and automatic face recognition (AFR) via the sensors of the user device, which are then analysed and used in the therapy. All elements are part of a speech therapy interaction, which can be applied in face-to-face and video therapy, as well as in asynchronous self-administered exercises. Following an initial requirements analysis [20], a co-creation development process was initiated, engaging people with speech and/or language impairment (PWSLI) and SLTs. The objective of this article is to evaluate the co-creation process in the HiSSS project to identify both opportunities and challenges in a co-created technology development process with PWSLI.

## Methods

The development process in HiSSS was based on the co-creation approach. The overarching objective of the development process was to facilitate collaborative work in a reciprocal partnership [50]. This encompasses the principles of open communication and mutual recognition of each other's expertise and forms of knowledge, thereby ensuring that all individuals can contribute meaningfully to the development process [21]. Furthermore, the endeavour aimed to mutual value creation, thereby ensuring that the technology itself would benefit, whilst concurrently empowering all participants to contribute their knowledge, skills and perspectives to the process [12].

For this development process evaluation, we have used the Guidance for Reporting Involvement of Patients and the Public (GRIPP2; [46]) reporting checklist to ensure the comprehensiveness of our reporting (Additional file 1).

### Overview

**Fig. 1:**
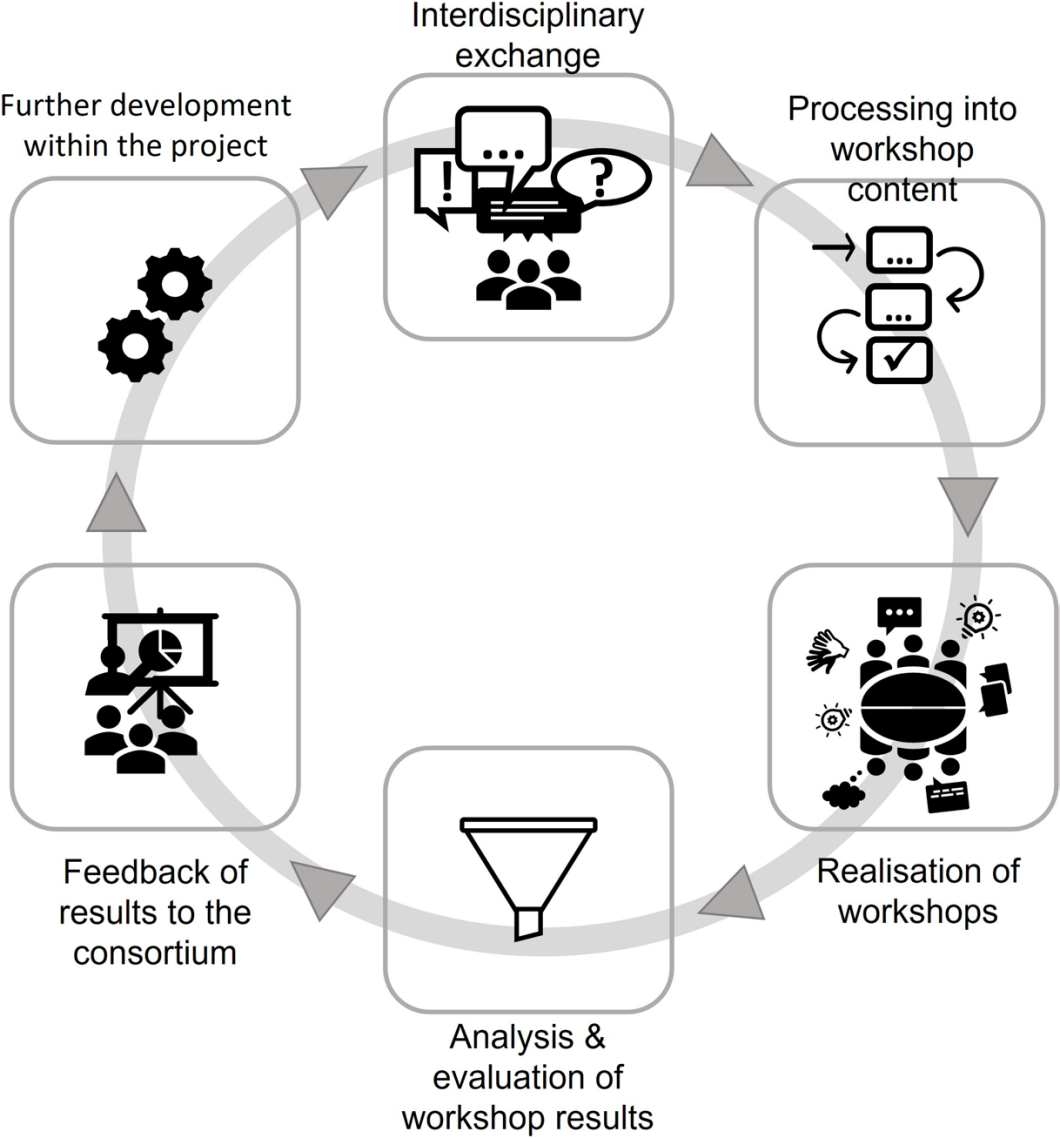
Workflow of the co-creation workshops in HiSSS

Co-creation workshops were integrated into the iterative development process of the project consortium. The consortium of the HiSSS project consists of four parties: two technology companies (Bitnamic, SpeechCare), a research institute for hearing, speech and audio technology (Oldenburg Branch for Hearing, Speech and Audio Technology HSA), and SLT research team at the Göttingen Health Campus with expertise in the field of speech and language therapy. The SLT research team was entrusted with user involvement during the whole development process. They were responsible for planning, organising, conducting, and evaluating the workshops. This division within consortia can also be found in other technology co-creation processes [18]. In order to align the content of the workshops with the state of development and to incorporate the results back into the development process, there was an exchange between the consortium partners prior to planning of the workshop content, as well as following the workshops. This resulted in the workflow shown in Fig. 1.

As Fig. 1 illustrates, the work process began with the consortium engaging in an interdisciplinary dialogue about the current status and current challenges for a new technology. They discussed the development steps for which user perspectives are needed. Based on this exchange, the SLT research team planned and conducted a workshop with SLTs and PWSLI. They then analysed the workshop and presented the results to the consortium. This exchange about the workshop results in turn led to new development ideas and plans.

### Co-creation workshops

The workshops were prepared, implemented, and evaluated by research-based SLTs. Workshops held with SLTs were conducted in a group setting, while an individual setting was chosen for workshops held with PWSLI based on previous research outcome and reflection [43]. A combination of both online and face-to-face workshops were organised in order to involve as many people as possible. Each workshop lasted 90 min. The workshops were planned in advance in the form of a manual. This included a timetable with the planned content and the materials required in each case. In some cases, questions and tasks were pre-formulated and adapted to the respective language level.

Certain arrangements based on the existing literature and the expertise of the facilitators, who are all SLTs, were made to involve the PWSLI and empower them to comment on the workshop content. Firstly, some basic communication principles were applied. These included the use of multimodal communication (verbal, gestural, writing, showing objects). To this end, the various options were presented to the co-researcher at the start of each workshop (chat, sending smileys and pictures, showing something to the camera). The co-researcher expressed their preferences among the different communicative possibilities and mainly chose the option of paraphrasing or asking questions. Chat was hardly used as a communication channel; gestures were mainly used in the face-to-face workshop and less in the online setting. Co-researchers were also offered the option of having a relative attend the workshop with them for support. This offer was accepted by two people. They consciously communicated where relatives were allowed to provide support. In advance, as well as in the workshop itself, the researchers discussed with the PWSLI and the relatives that support should only be provided as required by the PWSLI in order to avoid influencing or intrusive behaviour. Thus, the relatives only provided support within the workshops at the request of the respective PWSLI. In their own moderation, the moderators made sure to speak at a moderate pace and to adapt the language level so that it was comprehensible for everyone (short sentences, no foreign words).

Co-creation is the central methodological approach of the researchers in HiSSS to involve co-researchers in the development process of the teletherapy system. A range of methods were used to involve people in co-creation, e.g. brainstorming, experience prototyping, mind mapping, speculative design and observations. The methods used were tailored to the people and topics involved. A description of the methods used, including application examples, is shown in Table 1.

**Table 1:** Overview of the methods used in the co-creation workshops

Method	Definition	Example
Brainstorming	Brainstorming is a creative group technique aimed at generating a large number of ideas or solutions to a specific problem in a short time. It encourages participants to share ideas freely without fear of criticism, fostering an open and collaborative environment. Key principles include focusing on quantity over quality, welcoming unconventional ideas, and building on others' contributions [52]	Following the presentation of the contents of the teletherapy system to date, a brainstorming session was convened with the objective of identifying any content that had been omitted
Experience prototyping	Experience prototyping is defined as an approach to product development in which prototypes not only represent the functionality of a product, but also consider the overall user experience. In contrast to purely functional prototypes, which focus on the technical aspects, experience prototyping is oriented towards understanding how users interact with a product and the emotions that are triggered [55]	In one workshop, the co-researchers tested a prototype. While one co-researcher was engaged in testing the prototype, the others were observing his reactions. Subsequently, recommendations for enhancing the prototype were collected
Mindmapping	Mindmapping is a visual brainstorming technique used to organize information, ideas, or concepts around a central theme. It involves creating a diagram where the main topic is placed at the center, and related ideas branch out in a hierarchical or associative structure [25]	Mind mapping was used to collect solutions on how the teletherapy system can support home exercises. In a first step, the co-researchers note how they practise at home or how they instruct exercises at home. In a second step, they collected information on how technology can support this. For a limited period of time, these ideas were collected on a collaborative whiteboard so that co-researchers could respond to and build on each other's ideas
Speculative Design	Speculative design is an approach to design that explores future possibilities and scenarios rather than focussing on solving immediate problems. It uses design as a tool to imagine, question and explore possible futures, often pushing boundaries and challenging existing norms [19]	Co-researchers were tasked with the creation of a landing page of their choosing on a collaborative whiteboard. Co-researchers were invited to contemplate the attributes and configuration of a landing page that would be optimally effective. It was emphasised that the primary objective was not to consider the feasibility of the task, but rather to encourage co-researchers to engage in uninhibited associations and creativity
Prioritizing	Prioritizing is a decision-making method used to rank or order items based on their relative importance, urgency, or value. The process involves evaluating alternatives against specific criteria to identify the most critical elements for action or implementation [29]	Following the collective identification of the development and revision steps in a workshop, a prioritisation process was initiated to ascertain the relevance of each step
Observation	Observation is a research method used to systematically collect data by directly watching and recording behaviours, events, or phenomena in their natural context. It can be structured (with predefined categories) or unstructured (open-ended) [10]	Observation was used in a workshop for usability evaluation. Co-researchers were observed as they carried out predefined actions in the teletherapy system. It was observed whether they were able to solve the tasks and which solutions they tried

### Inclusion and exclusion criteria

Co-researchers included SLTs as well as PWSLI. One inclusion criterion for SLTs was that the individual must be a SLT who regularly treats speech and language impairments following a stroke. Additionally, they should have already gained experience with the implementation of teletherapy. The inclusion criterion for PWSLI was that they had a speech or language impairment as a consequence of a stroke or a similar neurological condition. No degrees of severity were deliberately excluded thus allowing all degrees to be represented. Experience with teletherapy was desirable but not essential. People diagnosed with depression or other psychiatric illnesses were excluded. The recruitment process was conducted through the collaboration of speech therapy practitioners, professional associations, and self-help groups. Contact was initially made in writing via email, with a project presentation and details of the co-creation workshops. Subsequently, a further contact was made by telephone, during which detailed and personal information was provided and potential queries were answered. All co-researchers provided written consent to participate.

The co-researchers were asked to complete a questionnaire on a number of socio-demographic factors (age, sex, education, occupational status). A diagnostic tool was initially employed to ascertain the severity of the speech impairment in PWSLI. On the one hand, this served to describe the co-researchers; on the other hand, it provided those carrying out the workshops with a detailed insight into the language profile of the co-researchers. This enabled the appropriate preparation and implementation of the workshops. The Bielefeld Aphasia Screening Rehabilitation (BIAS-R; [38]) was employed for individuals with aphasia, while the Bogenhausen Dysarthria Scales (BoDyS [56],) was used for those with dysarthria.

### Data collection

**Fig. 2:**
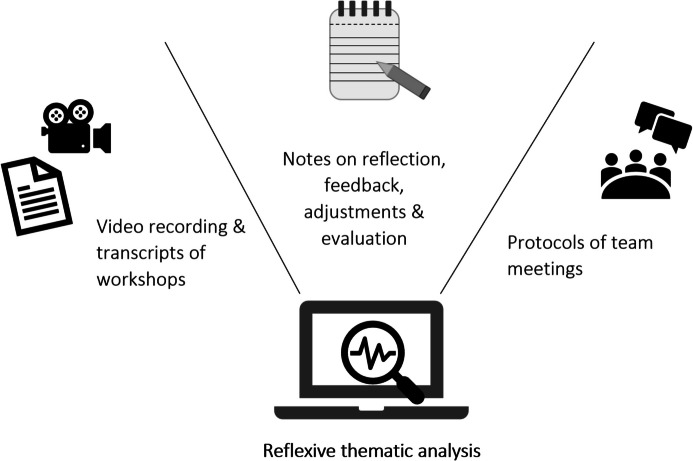
An overview of the data sources used

Various data sources were used as a basis for analysing the entire involvement process within this technology development. To facilitate analysis, the workshops were recorded on video and transcribed. The workshops spanned a period of 20 months (September 2022—May 2024), during which the perspectives of all co-researchers were to be integrated into the process. To this end, the workshops were designed to be iterative. In order to this end, the workshops were the subject of regular reflection and evaluation. The basis of this process was feedback from co-researchers and researchers' documentation with regard to the results achieved. The reflections, feedback and iterative adjustments that occurred during the development process were documented as notes and as such are also included as data material in this process analysis. Furthermore, the evaluation process incorporated the protocols of team meetings and the researchers' reflective notes, which were created during the course of the study. An overview of the collected data sources is given in Fig. 2.

### Data analysis

The data sources (workshop transcripts, process adaptations, team meeting protocols, researchers' reflective notes) were analysed using Braun and Clarke's reflexive thematic analysis (2006). The analysis process was guided by the overarching goal of process evaluation. Consequently, both challenges and opportunities were identified deductively. In a subsequent step, the authors generated inductive themes to which they assigned opportunities and challenges.

The content analysis was conducted by the first author (KG) who coded the material. Rigour was established through a process of ongoing debriefing and reviewing with the co-authors (MW, JL) to organize the findings and reach a consensus. Contextualization of the study findings serves to reinforce the credibility of the research. This entails the provision of comprehensive details regarding the context of the study, including information about the researchers and co-researchers, in order to ensure the transparent generation of study findings (McAllister & Lyons, 2022). The software MAXQDA 2022 for Windows as well as Microsoft Excel were used to manage the data during the analysis.

## Results

**Fig. 3:**
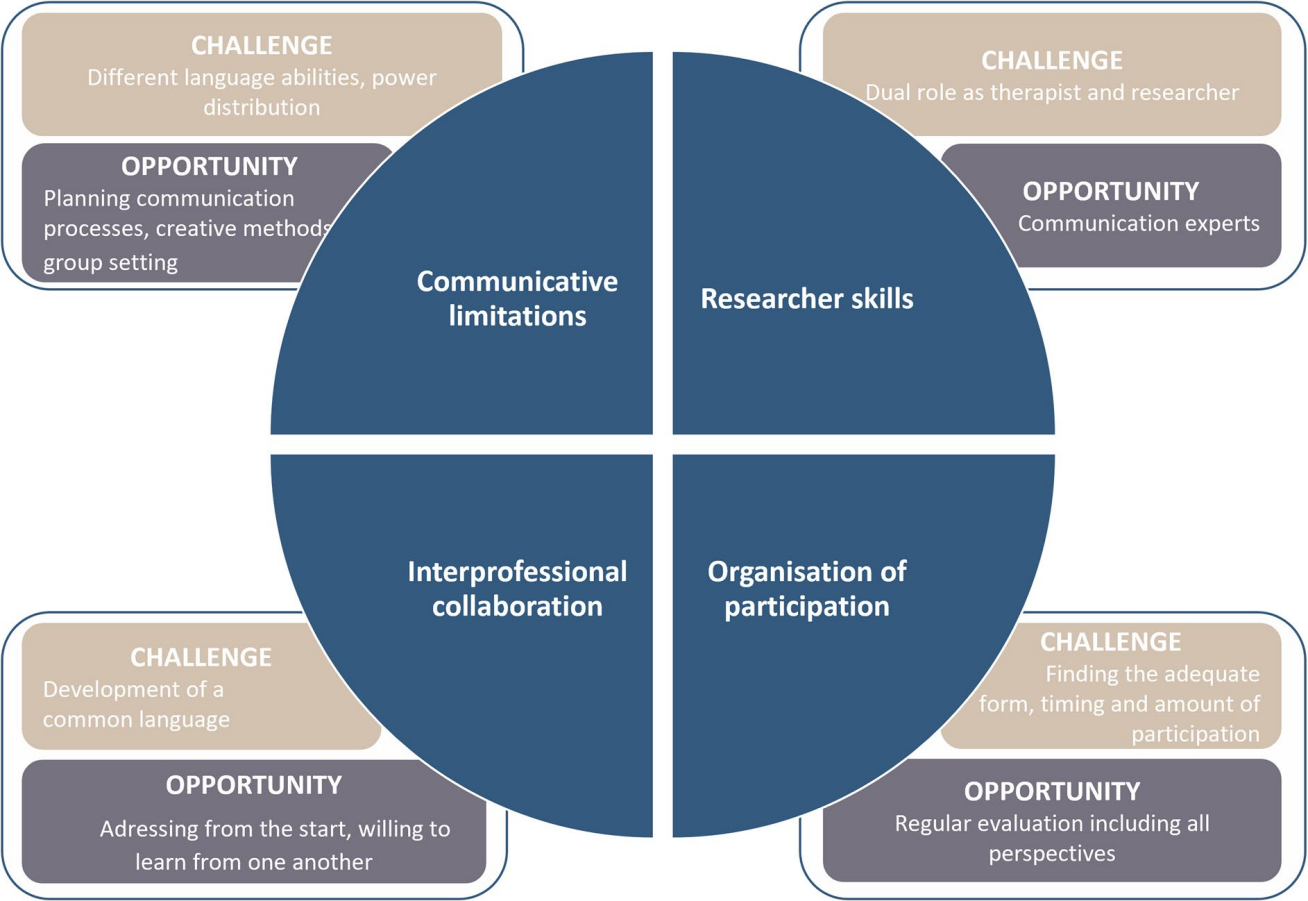
Four important themes for future technology co-creation with PWSLI and their challenges and opportunities

A total of six workshops with SLTs and four workshops with PWSLI were conducted. A total of four themes were generated from the available documents for the entire process: Communicative limitations; researcher skills; interprofessional collaboration; and organisation of participation. Each of these themes describes opportunities and challenges in relation to the development process of the teletherapy platform (Fig. 3). In this section, the context is first described in more detail, including the workshops that took place and the co-researchers involved. This is followed by a presentation of the four themes.

### Workshops

Table 2 provides an overview of the workshops, their setting, their content, and the number of co-researchers. Of the aforementioned workshops, only one was conducted in person, in a face-to-face setting. The remaining workshops were held remotely via video conference. The implementation of online workshops permitted the recruitment of co-researchers on a national scale, rather than a regional one.

**Table 2:** Overview of the workshops within the iterative development process

Workshops	Setting	Month/Year	Content	SLTs	PWSLI
1	Digital	09/2022	Initial feedback on a digital prototype	4	1
2	Digital	11/2022	Brainstorming on desired content (collect exercise ideas); brainstorming on customisable setting functions	2	
3	Digital	03/2023	Active testing of and feedback on the user interface	3	
4	Digital	06/2023	Developing user-friendly system views; brainstorming for motivating feedback	3	3
5	Digital	11/2023	Discussion of the objective feedback (diagrams & values)	3	3
6	F2F	05/2024	Usability testing & focus group to evaluate the system and requirements for instructions	4	4
			Total number of co-researchers throughout the process	11	7

*SLTs* Speech and language therapists, *PWSLI* People with speech and/or language impairment

### Co-researcher

A total of 11 SLTs participated in the co-creation workshops, as detailed in Table 3. All co-researchers met the inclusion criteria. All SLTs were female, with an average age of 31 years (SD of 10,4 years) and an average of 8 years (SD of 10,3 years) of professional experience. The majority of SLTs were in employment at the time of the workshops, with two having their own practice. The majority of SLTs attended one or two workshops, with one individual attending five of the six workshops. The majority expressed a desire to participate further and were subsequently invited to the following workshops. The most common obstacle to participation was the inability to attend the scheduled workshop date.

**Table 3:** Characteristics of the included SLT

	**Sex**	**Age (years)**	**Work experience (years)**	**Current status**	**Number of workshops attended**
SLT1	female	25	3.5	Employed	5
SLT2	female	58	35	Practice owner	2
SLT3	female	27	5	Employed	2
SLT4	female	46	22	Practice owner	2
SLT5	female	29	7	Employed	1
SLT6	female	25	3.5	Employed	2
SLT7	female	24	0.5	Employed	2
SLT8	female	26	2	Employed	1
SLT9	female	25	2	Employed	1
SLT10	female	29	7	Employed	1
SLT11	female	26	0.5	Employed	1

A total of seven people took part in the four workshops with PWSLI, comprising one woman and six men (see Table 4). The majority (6/7) was living with aphasia, while one had dysarthria. The severity levels were mild, moderate and severe, with a tendency towards mild and moderate. Almost half of the co-researchers attended more than one workshop. The primary rationale for this was the distinction between online and face-to-face settings. Those participating in the online workshops were unable to attend the face-to-face workshop due to distance.

**Table 4:** Characteristics of the included PWSLI

	**Sex**	**Age (years)**	**Speech or language impairment**	Severity *	**Number of workshops attended**
PWSLI1	male	57	Aphasia	mild	3
PWSLI2	male	54	Aphasia	severe	1
PWSLI3	male	63	Aphasia	moderate	2
PWSLI4	female	54	Aphasia	moderate	2
PWSLI5	male	75	Dysarthria	mild	1
PWSLI6	male	72	Aphasia	mild	1
PWSLI7	male	64	Aphasia	moderate	1

^*^ Measured with BIAS-R [38] or BoDyS [56]

### Communicative limitations

This theme encompasses all the facilitating and hindering aspects associated with the communicative limitations of the target group, PWSLI. Starting with the challenges, the different language abilities of the PWSLI have, have been a challenge for the interaction itself. This phenomenon can be illustrated by the observation that abstract content tended to be more challenging to communicate in comparison to concrete content. Therefore, as planned, visualisations were used for support. Another point was added by the PWSLI. Due to the various degrees of severity, the co-researchers showed different lengths of utterance and proportions of conversation. Some co-researchers expressed concern about the handling of turn-taking and taking up too much speaking time, as the following two quotes show.

*„So now I've also realised, well, you actually notice when you're talking, when you say it yourself, I've also noticed now that I've talked a bit too much, that was, and then the gentlemen don't talk so much.“* (PWSLI4)

*„You have to somehow, um, control yourself, don't you, so that you don't somehow, so to speak, um, steal time from others.“* (PWSLI1)

The moderators endeavoured to take this into account when moderating the workshops by specifically addressing people who showed stronger expressive impairments and offering more support to enable them to communicate.

Due to the disparity in language abilities between researchers and co-researchers, there was a risk of unequal power distribution. To avoid overburdening anyone linguistically during the workshops, a focal topic was chosen for each one. However, this meant that the co-researchers did not receive a coherent overview of the system as a whole and its development. Concurrently, they indicated a desire for a more comprehensive understanding of the overall system that was in development. Furthermore, there was a desire for greater transparency regarding the adjustments that had been made, specifically with regard to the development process. This demonstrates that, due to the selective involvement in HiSSS, the majority of the decision-making power was retained by the consortium. In response to the feedback received, efforts were made during the course of the workshops to provide co-researchers with an overview of the overall system. In the following workshops, the progress since the last workshop was first presented with the support of a few PowerPoint slides.

An opportunity that promoted the handling of the communicative limitations was, the prior planning of the communicative situation (as described in the Methods section). Despite these pre-planned adjustments, further steps were developed iteratively throughout the workshops. One methodological challenge for the moderators was to establish a creative and open working atmosphere. The concept of an open working atmosphere meant that co-researchers could interact with each other and contribute their own ideas with minimal influence from the moderators. The objective of the workshops was to elicit from the co-researchers the most authentic ideas and potential solutions for development topics of the presented technology. This essentially required minimal guidance, thereby facilitating the exercise of creative autonomy. In light of the communicative limitations, it was necessary to consider that openness also represents a significant communicative challenge. It is possible that the provision of support may facilitate linguistic expression, but it may also exert influence over the co-researchers in terms of content. This discrepancy between the desired level of creative openness and the potential for external influence was addressed through the iterative implementation of a staggered approach. The process typically commenced with the formulation of highly open-ended questions and the elicitation of spontaneous responses. Once the initial responses had ceased, more detailed follow-up questions could be incorporated. In some cases, impressions gathered in the open questions were also prioritised afterwards. The prioritisation could be supported visually (e.g. with smileys or numbers) so that all co-researcher could comment on it. Subsequently, the rationale behind the prioritisation could be explored through targeted inquiries. In order to vary the linguistic and cognitive demands within a single workshop, generative methods (idea generation, e.g. common understanding of …, solutions for …) and evaluative methods (e.g. evaluation of prototypes) were always combined and alternated [54]. Additionally, the combination of methods allowed co-researchers to engage in the process in different ways.

Furthermore, the group setting provided an additional opportunity for dealing with communicative limitations. The configuration of workshops with PWSLI was subject to adaptation. Following the initial workshop, which was conducted on an individual basis for the PWSLI, the researchers determined that group dialogue would be conducive to creative work. Consequently, the ensuing workshops were conducted in small groups. The number of co-researchers per workshop ranged from one to four, with the majority of workshops comprising three individuals. In the group setting, the co-researchers benefited from hearing the ideas of others and thus being able to either agree or disagree with ideas or to build on them and make their own additions. In some cases, they needed less linguistic effort for this than for presenting their own thoughts in a one-to-one setting. At the same time, this group size was suitable for addressing the needs of everyone involved and their communication impairments.

### Researcher skills

The preceding theme has demonstrated the necessity for researchers to consider PWSLI when conducting their research. This theme describes the role ambiguity that the researchers experienced due to their different competences. During the SLT researchers' team meetings, one challenge that emerged was that they sometimes experienced a conflict of roles when reflecting on the workshops. On the one hand, they are accustomed to their role as therapists. In this capacity, they provide empathic support and specialist knowledge about PWSLI, as well as corresponding strategies for supporting communication. On the other hand, as researchers, they adopt an open attitude and refrain from influencing the co-researchers. While this restraint makes sense in research, it can reach its limits when dealing with PWSLI who need support with communication. It was realised that the roles of SLT and researcher are equally embodied, and that the SLT researcher moves back and forth between them with regard to the goal of enabling the participation of PWSLI.

Their competences as SLTs proved to be an opportunity for the researchers. It is imperative that expertise in dealing with communicative impairments is employed in order to successfully involve PWSLI as active partners. Speech and Language Therapists (SLTs) are experts in this field and are familiar with the use of language support strategies. This phenomenon was particularly evident during the interdisciplinary exchange. Technology partners from the consortium found it much more difficult to assess and deal with the linguistic abilities of PWSLI. For example, one computer scientist reported in a team meeting after a workshop that he had noticed himself becoming impatient due to the slow speech or repetitions of the PWSLI. The existing expertise of the SLTs in turn took the pressure off him to react.

### Interprofessional collaboration

Interprofessional collaboration was generated as a third theme. From the outset, the project was conceived and planned in an interdisciplinary manner. Different areas of expertise were considered during the planning stage, and areas of responsibility were allocated accordingly. However, the challenge of synchronising the different ways of thinking and working was underestimated by everyone involved. During the first year of the project in particular, different interpretations of individual terms often became apparent during team meetings. This initially led to misunderstandings and was quite time-consuming to resolve. Explaining terms in their respective disciplinary contexts helped to uncover differences.

In the second year of the project, it became apparent that a further challenge was to ensure that common goals were not lost sight of. The consortium partners pursued different sub-goals according to their areas of expertise. However, when focusing on these sub-goals, the common goal sometimes faded into the background. This was exacerbated by the division of tasks depending on expertise, resulting in processes running separately from one another. One example of this is the organisation of the workshops. In the first workshops, the SLT research team was solely responsible for preparing, running, and evaluating the workshops. It became clear that integrating the results into the development process was only partially successful. This was reflected in the limited consideration given to the results of the workshops, and thus the feedback from the co-researchers, in the technical development.

Opportunities for successful interprofessional collaboration were identified in the course of the process. These can be summarised in the willingness to learn from one another. One opportunity is for technicians and co-researchers to exchange their respective technical and everyday expertise.

Because of this technical consortium partners also took part in the workshops, depending on the topic, from Workshop 4 onwards. The added value of this was that they were able to hear the feedback and ideas of the co-researchers unfiltered, to respond with their expertise, and to engage in dialogue. The consortium partners' participation in the workshops enabled them to engage in interprofessional dialogue, thereby enhancing their capacity to identify potential workshop topics. While the speech therapy research team retained primary responsibility for planning, organising, and evaluating the workshops, the input of other consortium partners was also represented.

Also, the evaluation process was therefore modified, so that the results were presented in the form of possible consequences and work packages. This facilitated an integration of the results into the subsequent stages of development. Furthermore, smaller, more focused meetings were preferred for workshop outcomes that were of particular interest to individual consortium partners, as opposed to convening a large meeting with all parties. In our experience, this approach facilitated a goal-oriented exchange.

With regard to the synchronisation of project goals, the open and regular exchange in team meetings proved to be helpful. Individual sub-goals were sometimes discussed in team meetings, helping to bring the overall consortium together on the basis of their common end goal, despite specific expertise. Furthermore, it became apparent that the different disciplines could benefit from each other's perspectives. For instance, when presenting technical implementation plans, the SLT research team could appropriately evaluate the therapeutic relevance of certain technical options. Meanwhile, the technical partners could assess various implementation options based on feasibility and cost.

### Organisation of participation

The fourth and final theme, which was created through the analysis, outlines the challenges and opportunities that arose during the organisation of the entire participation process. As the previous themes have shown, the organisation of participation in the form of co-creation workshops evolved over the course of the process. This was due to regular exchange formats within the entire consortium or individual parts. Finding the adequate form, timing and amount of participation proved difficult in these exchanges. The reasons for this were different expectations and ideas on the part of the consortium regarding the workshops, different perspectives on the workshop participants (SLT and PWSLI), and different working methods (e.g. agile development versus scientific analyses). This became particularly clear at a consortium meeting that took place after the first two workshops. During this meeting, the entire consortium came together in person for two full days. This time was used, among other things, to collaborate on answering the question, 'How can participation succeed?'. This revealed different perspectives and requirements for participation, such as equal communication and coresearchers acting as test users.

After identifying these different perspectives, the opportunity arose to jointly reflect on and adapt the participation process. Then, the consortium reflected on how the previous workshop organisation had worked, considering what had met their expectations and what had not. This discussion was moderated by someone external to the project, which had a very favourable effect, as it ensured that all consortium partners were equal participants.

In addition to the regular reflection sessions held within the consortium, co-researchers were asked at the end of each workshop to share their thoughts on their experience and suggest ways in which it could be improved. Overall, the co-researchers gave positive feedback about their experience. They emphasised in particular the opportunity to help shape the project, and the sense of meaningfulness and value they gained from the experience.

*„I think it's great, especially when we're together, so you're a small part of something good“* (PWSLI6)

Above all, the co-researchers wanted to be involved in the long term so that they could follow further progress and development steps.

*„I don't know if it's feasible if the group that is working here now, yes? If, when it's ready, they simply get some information, an exchange like that, that might also be useful“* (PWSLI5)

## Discussion

Digital technologies represent an opportunity in speech and language therapy to improve access to therapy and also the frequency of therapy (Cason et al., 2014; [34]). However, these benefits only arise if the technology takes into account the actual needs of the target group and is adapted to them. Involving representatives of the target group can help to achieve this goal. It can also have positive effects for those involved, who can play an active role. However, these positive effects do not materialise automatically. For instance, the co-researchers in HiSSS perceived their participation as positive and valued their involvement. At the same time, they criticised the fact that they were given too little overview of the overall system. However, such an involvement process is very complex in itself. For this reason, it is important to evaluate participation processes to identify areas for improvement in future. For the HiSSS project, we conducted a thematic analysis according to Braun & Clarke [6] to identify the challenges and opportunities within the development process. Four themes were generated to which these could be assigned. These represent dimensions of co-creation that should be considered in future co-created technology developments. The following discussion will proceed by exploring the four dimensions in the context of further literature.

### Communicative limitations

One of the most crucial prerequisites for a technology co-creation development process is always the exchange between the co-researchers and the development team, which can be defined as communication (Daly-Lynn et al., 2016). Consequently, in the context of PWSLI participation it is necessary to identify methods that facilitate communication. This is in accordance with recent published research on approaches to conducting interviews [36]. It is essential to plan the communication processes in advance. This includes considering the co-researchers and their language profile, as well as preparing possible support materials and offering different communication channels. The selection of methods also has an influence on the opportunities for participation. The deliberate selection of participatory research methods, tools, and processes can help researchers in involving co-researchers in the research process, which in turn has the potential to create relevant, meaningful research results that are subsequently implemented for further development of the technologies (Vaughn et al., 2020). For technology development in speech and language therapy, this means adapting existing procedures and using complementary methods that are tailored to the specific requirements of PWSLI [44]. Furthermore, the use of creative methodologies, whereby communication is conveyed through actions, can prove beneficial [32]. Although it can be easier to adapt to the individual needs of people in a one-to-one setting, our results have demonstrated that the group setting offers considerable advantages in this process. In a group setting, co-researchers can engage in communicative interaction, building upon the statements of others. Furthermore, the content is enhanced by this approach, as it facilitates the generation of more detailed data through the exchange and discussion of ideas. In addition, a shared team atmosphere is created, which can have a positive influence on the experience [32]. However, this confirms that it is not the severity of an impairment that is decisive for successful involvement, but the type of conversation that is offered [26].

Ultimately, it is also important to consider the potential for unequal distribution of power when linguistic abilities differ. Unequal distribution of power refers to the imbalance in decision-making authority, influence and control between researchers and co-researchers [32]. The risk of unequal distribution is higher when working with people with communicative limitations [32]. Due to their limitations, they can be misunderstood or they can express themselves less clearly and are given less consideration than people without communicative limitations. In addition, they are sometimes dependent on the support of others, which can unintentionally restrict their autonomy and distort the distribution of power [40]. In HiSSS, the majority of decision-making power was retained by the consortium due to constraints on resources. In the future, it should be investigated how to achieve equal partnership in collaborative projects with PWSLI while respecting their resources [47].

### Researcher skills

During the process, it became evident that adequate involvement of PWSLI requires moderators who possess training in the field of communication, in order to ensure a successful conversation. For the researchers, this implies a need to understand PWSLI and be able to interact with them using appropriate questioning techniques and communication methods. As in previous projects, SLTs with research experience proved to be a suitable choice in HiSSS [36]. However, the reflection process also revealed a potential conflict of roles for the therapists engaged in the research. While they respond empathetically in their role as speech-language therapists and employ elements of supported communication, as researchers they must maintain an objective openness and refrain from making contributions of their own. It is also known from other professions, such as occupational therapy or psychotherapy, that the roles of ‘therapist’ and ‘researcher’ are associated with different competences and responsibilities [22, 45]. The term 'role ambiguity' has been used in relation to the increasing scope of speech and language therapy and the subsequent emergence of new specialisms (ASHA, 2009). It is intended to describe uncertainty about professional boundaries and overlaps with other professions. Role ambiguity could also be applied to the situation experienced by the SLT researchers in HiSSS. The uncertainty lies in the extent to which they demonstrate therapeutic competence and apply research-related skills. Successful integration of PWSLI requires a thoughtful balance of competences from both areas.

It is therefore important to reflect regularly on whether it is possible to provide support without exerting influence. Consequently, SLT researchers must be aware of their own dual role as practitioners engaged in the research process.

### Interprofessional collaboration

In many technology co-creation projects with PWSLI, other disciplines will be involved in addition to the research-based SLT. The results and reflections described highlight obstacles in an interprofessional co-creative process. On the one hand, the involvement of multiple disciplines is a fundamental aspect of a technology co-creation process. On the other hand, it can also be a challenge. It is essential to recognise this challenge and address it proactively in order to overcome it collectively. Altizer et al. [1] identify the need to develop a common understanding as the greatest challenge for interprofessional collaboration. This obstacle should be recognised and addressed at the start of a project to prevent misunderstandings later on. The prerequisite for this is that all members of the interprofessional team learn to communicate with each other and are willing to learn from one another [1]. Integrating the different perspectives can be a challenging process, as they are often intertwined and able to influence one another reciprocally [24]. In a technology co-creation development process, interprofessional cooperation should also include working with the practice partners involved. The involvement can be organised primarily by one partner, but the other partners should also take part in the work phases, such as our co-creation workshops, in order to achieve a true co-creation process.

### Organisation of participation

Against the background of the above-mentioned aspects, a technology co-creation development process should be regularly reflected upon and evaluated. This evaluation should include the perspectives of all those involved. The perspective of the co-researchers is particularly important, as involvement can also be perceived as too much [5]. This shows that the level and timing of participation must be chosen carefully, for while participation is essential, more is not always better, especially with a vulnerable group [11]. When planning the timing and scope of participation, its added value must therefore be compared with the resources involved [48]. This shows the contextual and individual factors of technology co-creation processes, highlighting the relevance of their evaluations.

### Strength and Limitations

The difficulty in recruiting co-researcher was a significant factor in the project's outcome. The recruitment of PWSLI in particular proved to be particularly challenging, which is why the first workshop was held with only one individual. Although we endeavoured to include a wide range of severity in the PWSLI, it was predominantly people with a moderate or mild impairment who took part in the workshops. In particular, the co-researchers' language comprehension was only slightly affected. Process analyses of involvement processes with people who are more severely impaired should be tailored by future studies. If co-researchers with health issues are involved in research or a development process, it is important to clearly communicate the intention of participation. In HiSSS it was emphasised from the outset that the aim was to collaborate on a technology development and that there would be no language-specific intervention. However, subsequent conversations revealed that the co-researchers with speech and language impairments were still hoping for an improvement in their language skills by participating in a project with SLT researchers. Addressing this expectation of improvement is an ethical issue that requires transparency from the outset and throughout the project.

Another aspect of co-creation processes that requires critical consideration from an ethical perspective is the technological expectations of the co-researchers. Working on a new technology can create hope and expectation, which may lead to frustration if a finished, market-ready product is not created by the end of the project [16]. From the outset, the co-researchers should be made aware of the goal and the timeframe for the delivery of a final product.

All of the participating therapist co-researchers were female. This is not unusual compared to the wider profession, which is also predominantly female (Litosseliti & Leadbeater, 2012). Therefore, no bias is expected from this distribution in the data for this study. A strength of this study is the early involvement of the co-researchers. Unlike many studies, where co-researchers are only involved in later project phases for evaluation and testing [33], in this study they were involved from the conceptualisation and design stages. The authors acknowledge that their background as speech and language therapists and their own involvement in the development process could have introduced potential biases in both the data collection and the analysis of the study.

## Conclusions

This paper demonstrates the complexity of technology co-creation with people with speech and language impairments. Building on previous research, it confirms that people with speech and language impairments (PWSLI) can be effectively involved in research and contribute meaningfully to technology development. The success of their involvement does not depend on the severity of their impairment, but rather on the conversational opportunities provided [26]. However, addressing language characteristics is only one of the four identified dimensions. Additionally, specific skills are needed for researchers, and interprofessional collaboration must be functional. Phases for reflection and adaptation must also be planned and considered from the outset. These dimensions are of particular importance for ensuring sustainable use of the technology after the research project has been completed, with the aim of preventing possible discontinuation of use and increasing the likelihood of acceptance of the new technology. In order to take these four dimensions into account in future co-creation projects, the funding conditions must provide the necessary framework. Funded projects should be able to respond quickly to reflection on processes and adapt them. Resources must be allocated to facilitate constructive interprofessional collaboration and to integrate training opportunities on involvement methods and the necessary self-reflection for researchers [28].

It is possible to involve PWSLI in development processes successfully. Nevertheless, an approach is necessary that is methodologically designed and oriented to the target group. It is essential to ascertain, at the earliest possible stage and on a case-by-case basis, whether and to what extent users should be involved in research projects, in accordance with the specific objectives of the project in question. It is only then that sufficient resources can be allocated in order to fulfil the qualitative and ethical requirements of user involvement.

## Supplementary Information

Additional file 1.

## Data Availability

No datasets were generated or analysed during the current study.

## Abbreviations

SLT: Speech and language therapist
PWSLI: People with speech and/or language impairment
HiSSS: Hybrid and Interactive Speech and Language Therapy after Stroke
GRIPP: Guidance for Reporting Involvement of Patients and the Public
