# Supplementary material for: The involvement of individuals with speech and language impairments in research: insights from a co-creation process
Source: Res Involv Engagem. 2025 Aug 4;11:91. doi: 10.1186/s40900-025-00762-8 (PMC12320304; doi:10.1186/s40900-025-00762-8)
Supplement: Supplementary file 1 — Additional file 1. [file 40900_2025_762_MOESM1_ESM.docx]

| Section and topic | Item | Reported on page/line |
| --- | --- | --- |
| **Section 1: Abstract of paper** |  |  |
| 1a: Aim | A comprehensive look at the co-creation process in the HiSSS project will highlight opportunities and challenges for a successful and sustainable implementation of technologies in the speech and language therapy process. | Page 2  Lines 40-42 |
| 1b: Methods | The data sources (workshop transcripts, process adaptations, team meeting protocols, researchers' reflective notes) from the co-creation process were analysed using Braun and Clarke's reflexive thematic analysis (2021). Both challenges and opportunities were identified deductively through the analysis. In a further step, the authors inductively generated themes to which they assigned the opportunities and challenges. | Page 2  Lines 44 - 48 |
| 1c: Results | Six workshops with a total of 11 speech and language therapists and four workshops with a total of 7 people with speech and/or language impairments took place. The methodological adjustments for the integration of PWSLDs are described in detail. The implementation revealed four key aspects that should be taken into account in the planning and implementation: (1) Communicative limitations, (2) Researcher skills, (3) Interprofessional limitations, (4) Organisation of participation. | Page 2  Lines 50-53 |
| 1d: Conclusions | It is possible to successfully integrate PWSLD into development processes. However, an approach is necessary that is methodologically designed and oriented to the target group. Whether and to what extent users should be involved in research projects must be clarified as early as possible and on a case-by-case basis according to the specific objectives of the respective project. This is the only way to provide sufficient resources to fulfil the qualitative and ethical requirements of user participation. | Page 3  Lines 55-61 |
| 1e: Keywords | Co-Creation, Involvement, Speech and Language Therapy, Stroke | Page 3  Line 65 |
| **Section 2: Background to paper** |  |  |
| 2a: Definition | The core idea of all these approaches is to involve stakeholders and thus view the potential end users of objects as the subjects of the development process, with equal and reciprocal relationships between professionals and end users (D'Haeseleer et al., 2021; Halvorsrud et al., 2021). Participation can vary by type and time. This allows participants to take on different roles from listener to decision-maker (Smits et al., 2020), which can be applied throughout the research process or at specific stages. | Page 5  Lines 113-118 |
| 2b: Theoretical underpinnings | The communication between all those involved is an important component in order to enable innovation, share knowledge, and promote acceptance (Daly-Lynn et al., 2016). This becomes a challenge for groups of people with limited language-based skills due to their impairment. | Page 5  Lines 118-121 |
| 2c: Concepts and theory development | Singh et al. (2022) identify potential strategies to overcome limitations in the inclusion of people with stroke, especially those with communication disorders. These include involving speech and language therapists (SLTs), using generative tools to enable participation (e.g. Lego serious play, videos, mapping methods), and assessing communication profiles in order to be able to respond to them individually. These strategies can be found in co-design examples with people with communication disorders. The involvement process is typically conducted by or with the assistance of SLTs, and both spoken and written material are adapted to the linguistic abilities of the involved individuals (Anemaat et al., 2024; Avramović et al., 2023; Spelter et al., 2022). Furthermore, the use of visualisation techniques to enhance comprehension and focus has been demonstrated to be beneficial (Avramović et al., 2023; Pierce et al., 2024; Spelter et al., 2022). There is a consensus that sufficient time and resources should be allocated to address the communicative needs of individuals with communication disorders, whether through additional training or support offers (Anemaat et al., 2024), the organisation of individual meetings (Spelter et al., 2022), or the option of confirming one's own statements in the form of member checking (Avramović et al., 2023). | Pages 5-6  Lines 126-136 |
| Section 3: Aims of paper | The objective of this article is to evaluate the co-creation process in the HiSSS project to identify both opportunities and challenges in a co-created technology development process with PWSLI. | Page 7  Lines 161-162 |
| **Section 4: Methods of paper** |  |  |
| 4a: Design | See section “Methods” | Pages 7  Lines 164-171 |
| 4b: People involved | A total of 11 SLTs participated in the co-creation workshops, as detailed in Table 2.  A total of seven people took part in the four workshops with PWSLD, comprising two women and five men (see Table 3). | Page 13  Line 289  Pages 14  Lines 298 |
| 4c: Stages of involvement | See Table 1 “Overview of the workshops within the iterative development process” | Page 12-13  Line 286 |
| 4d: Level or nature of involvement | The workshops were prepared, implemented, and evaluated by research-based SLTs. Workshops held with SLTs were conducted in a group setting, while an individual setting was chosen for workshops held with PWSLD based on previous experience (Spelter et al., 2022). A combination of both online and face-to-face workshops was organised in order to involve as many people as possible. Each workshop lasted 90 minutes. The workshops were planned in advance in the form of a manual. This included a timetable with the planned content and the materials required in each case. In some cases, questions and tasks were pre-formulated and adapted to the respective language level. | Pages 8-9  Lines 194-223 |
| **Section 5: Capture or measurement of PPI impact** |  |  |
| 5a: Qualitative evidence of impact | Not applicable |  |
| 5b: Quantitative evidence of impact | Not applicable |  |
| 5c: Robustness of measure | Not applicable |  |
| **Section 6: Economic assessment** | Not applicable |  |
| **Section 7: Study results** |  |  |
| 7a: Outcomes of PPI | Not applicable |  |
| 7b: Impacts of PPI | Overall, the co-researchers gave positive feedback about their experience. They emphasised in particular the opportunity to help shape the project, and the sense of meaningfulness and value they gained from the experience. | Page 20  449-451 |
| 7c: Context of PPI | See section “Overview” | Page 7  Lines 176 - 192 |
| 7d: Process of PPI | A total of four themes were generated from the available documents for the entire process: Communicative limitations; researcher skills; interprofessional collaboration; and organisation of participation. See section “Results” | Pages 12 - 20 |
| 7ei: Theory development | Not applicable |  |
| 7f: Measurement | Not applicable |  |
| 7g: Economic assessment | Not applicable |  |
| **Section 8: Discussion and conclusions** |  |  |
| 8a: Outcomes | For the HiSSS project, we conducted a thematic analysis to identify the challenges and opportunities within the development process. | Page 20  Lines 466 - 471 |
| 8b: Impacts | Four themes were generated to which these could be assigned. These represent dimensions of co-creation that should be considered in future co-created technology developments. | Page 21  Lines 471-474 |
| 8c: Definition | Not applicable |  |
| 8d: Theoretical underpinnings | “This is in accordance with recent published research on approaches to conducting interviews (Prior et al., 2020).”  “Ultimately, this confirms that it is not the severity of an impairment that is decisive for successful involvement, but the type of conversation that is offered (Lee & Azios, 2020).”  “It is also known from other professions, such as occupational therapy or psychotherapy, that the roles of ‘therapist’ and ‘researcher’ are associated with different competences and responsibilities (Hinojosa, 2003; Spengler & Lee, 2020). (…)” | Page 21  Lines 479-480 |
| 8e: Context | Not applicable |  |
| 8f: Process | See section “Discussion” and its subchapters:  “Communicative limitations”, “Researcher skills”, “Interprofessional collaboration” and “Organisation of participation” | Pages 20-25 |
| 8g: Measurement and capture of PPI impact | Not applicable |  |
| 8h: Economic assessment | Not applicable |  |
| 8i: Reflections/critical perspective | See section “Strength and limitations” | Page 24  Lines 553-580 |
